# Dimension Control of Hexagonal SiGe Single Branched Nanowires

**DOI:** 10.1021/acs.nanolett.5c00267

**Published:** 2025-03-26

**Authors:** Denny Lamon, Hidde A. J. van der Donk, Marcel A. Verheijen, Marvin M. Jansen, Erik P. A. M. Bakkers

**Affiliations:** † Department of Applied Physics, Eindhoven University of Technology, 5600 MB Eindhoven, The Netherlands; ‡ Eurofins Materials Science Netherlands BV, 5656 AE Eindhoven, The Netherlands

**Keywords:** nanowires, branches, hexagonal, Si, Ge, growth rate, heterostructure

## Abstract

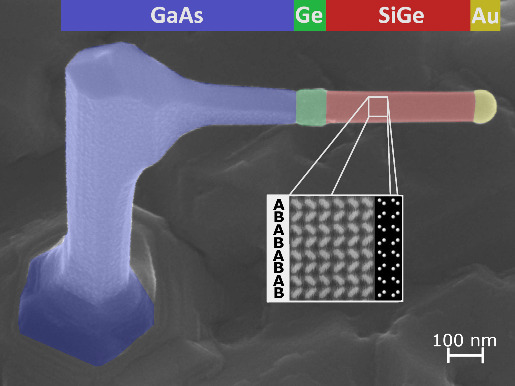

Hexagonal SiGe, with its direct band gap, holds promising light-emission properties and potential for advanced optoelectronic applications. The growth of this material has been achieved as nanowires, within core–shell or multibranch trunk structures. However, core–shell designs are limited to radial growth, restricting the axial dimensional control, while multibranch structures lack growth precision, reducing their practical applicability. Here, we introduce a novel technique to grow hexagonal SiGe as single-branched nanowires, achieving unprecedented control over dimension and morphology. The branch diameter is precisely tuned by adjusting the trunk diameter, leveraging the use of the same Au catalyst particle throughout both trunk and branch growth. We investigate the growth rate and its diameter dependency within the Gibbs–Thomson framework, providing valuable insights into growth dynamics. This innovative method opens new opportunities for advanced studies on hexagonal SiGe, paving the way for developing next-generation quantum devices.

In the semiconductor industry, Silicon (Si) and Germanium (Ge) have dominated the manufacturing and production processes due to their abundance, low cost, and well-established fabrication techniques. They have largely dominated the production of transistors, microchips, solar cells, and other electronic devices.

However, unlike group III–V compound materials such as gallium arsenide (GaAs), Si and Ge, in their most common diamond cubic crystal form, zincblende (ZB), exhibit an indirect band gap, which makes them unsuitable for optoelectronic applications.

Light-emitting Si would enable the seamless integration of photonics and electronics on the same chip, simplifying the fabrication processes and reducing costs. Moreover, faster communication speeds with lower power consumption could be accomplished.

Recent advances^1^ have revealed that Ge and Si_1–*x*_Ge_*x*_ alloys can exhibit direct band gap properties when crystallized in a metastable hexagonal 2H structure. This phase allows for a direct band gap down to a Ge content of approximately 65%, opening up new possibilities for their use in optoelectronics. III–V/Si-Ge core/shell nanowires (NWs) have been the template for the fabrication of 2H group IV materials for almost one decade,^2,3^ enabling the realization of complex quantum structures, including two-dimensional hexagonal SiGe (hex-SiGe) quantum wells^4^ with promising optoelectronic properties.

In addition, 1D nanostructures have been explored using a III–V/-Si–Ge trunk/branch growth technique,^5^ guaranteeing the production of pure hex SiGe NWs with potential in light emission and quantum applications. However, this fabrication method faces several challenges, particularly in controlling the morphology of the nanobranches. The ability to precisely tune the diameter of these branches, a critical factor for achieving quantum confinement, remains limited, and the growth direction and number of branches are often random, posing challenges to uniformity in device fabrication.

In this paper, we introduce a promising approach for in situ growth of single hexagonal SiGe branched nanowires with length and diameter control at the nanometric scale. Unlike traditional methods that utilize Au catalyst deposition on the nanowire side facets, we adopt a more efficient technique by employing the same Au particles to first catalyze the growth of a GaAs trunk, followed by SiGe branch growth, using the vapor–liquid–solid (VLS) mechanism in a metal–organic vapor phase epitaxy (MOVPE) reactor. Between the trunk and branch growth phases, the Au catalyst undergoes inflation and destabilization, following the method proposed by Tornberg et al. with InAs nanowires.^6,7^ High-quality III–V/IV materials heterointerfaces have been obtained, and a systematic investigation of the role of surface energies and the Gibbs–Thomson effect on the nanowire growth process has been carried out.

Hexagonal SiGe branches are grown on the side facets of wurtzite GaAs nanowires. In Figure 1a the key steps of the growth process are schematically illustrated.

(1) Au catalyzed hexagonal GaAs NWs with induced pyramid are grown in the [0001]_B_ direction from a GaAs (111)_B_ substrate, using Trimethylgallium (TMGa) and Arsine (AsH_3_) as precursor gases in the MOVPE chamber.^1^ The formation of pyramids is intentionally promoted through the inclusion of ZB segments that locally enhance the lateral growth rate (explored in detail in the Supporting Information (SI)). This reduces the occurrence of broken wires during the inflation of the Au droplet with Ga.
(2) The catalyst particle is destabilized by Ga accumulation, induced by switching off the AsH_3_ flow. The high growth temperature of 615 °C ensures the Au–Ga compound remains in the liquid phase, allowing significant Ga incorporation.^8^ The particle is therefore inflated, causing it to wet the side facets of the trunk.
(3) The particle is deflated again by introducing only AsH_3_ precursor for 5 min, reducing the catalyst volume and forming a flag-shaped structure growing in the  directions. Following this, an equilibration step with a V/III precursor ratio of 2.4 is carried out to complete the GaAs segment’s structure. Finally, a 4 min AsH_3_ step is performed at the end of the GaAs growth to obtain a flat solid–liquid interface as will be discussed in more detail.
(4) After cooling the sample, the reactor is flushed with hydrogen to remove precursor residuals. The growth temperature is adjusted to 420 °C for the growth of a hexagonal lattice matched Ge segment using germane (GeH_4_) as a precursor.
(5) The branch is finally extended with a Si_0.5_Ge_0.5_ segment grown after a 5 min temperature ramp up to 436 °C, with Disilane (Si_2_H_6_) introduced to achieve a precursor ratio of GeH_4_/(Si_2_H_6_ + GeH_4_) = 0.8.

**Figure 1:**
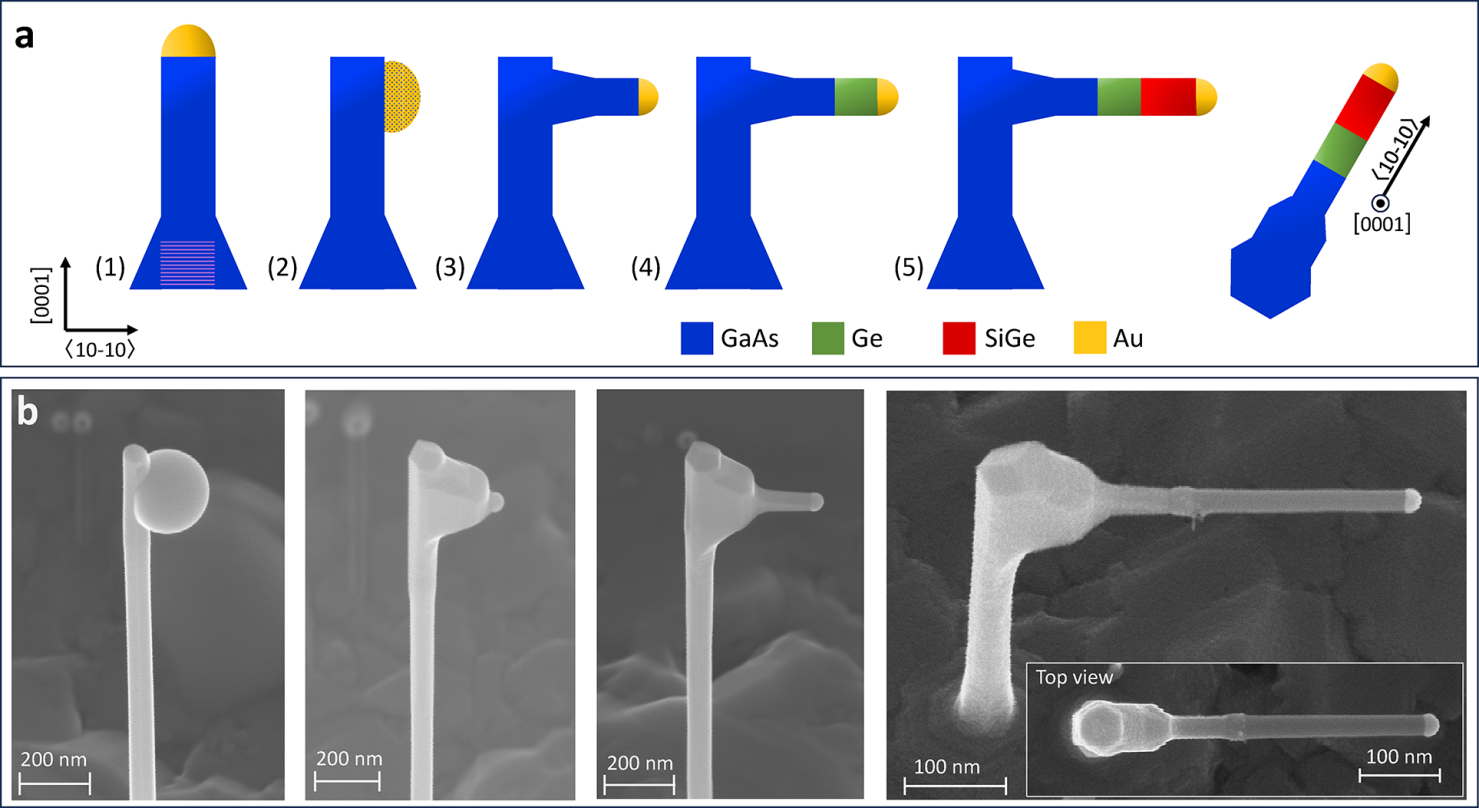
a) Schematic of the primary growth steps, from the GaAs nanowire trunk to the SiGe branch, including the main view directions. b) SEM micrographs of the growth history of a single branch, acquired at a tilted view at 30°. The inset image at the bottom right shows the complete structure from a top-view perspective.

As depicted in the scanning electron microscopy (SEM) images in Figures 1b, straight, untapered branches of approximately 400 nm in length are grown perpendicularly from the GaAs trunk in the  directions. High-resolution high-angle annular dark field (HR-HAADF) scanning transmission electron microscopy (STEM) imaging, shown in detail in the following, confirms the hexagonal crystal structure and the successful epitaxy at the GaAs/Ge/SiGe heterostructure. The multiple steps involved in the growth process inevitably affect the overall yield of the wires. Despite inducing the pyramids at the base, the yield of standing GaAs wires after the destabilization process (2) is diameter-dependent and is currently around 60–70% of the deposited Au seed particles. Five fields, each containing 20 by 20 Au particles were analyzed for statistical purposes. However, inconsistencies are present due to unavoidable intrasample variations. Further reductions in yield occur during steps (3) and (4) as well, resulting in a final success rate of approximately 10% high-quality wires. The final growth step of SiGe on Ge (5) is generally successful. Additional data are provided in the SI. Although yield optimization is beyond the scope of this work, several approaches could be explored to achieve full control over nanowire growth. Enhancing the thickness and spacing of zincblende inclusions at the base could lead to thicker base pyramids, improving the nanowires’ survival rate. Likewise, using an alternative trunk material, such as GaP or InAs, may be more energetically favorable for the growth of straight branches.

The use of the same Au catalyst particle for the growth of both the trunk and the branch is expected to offer a significant advantage in controlling the diameter of the branches. The relation between the diameter of the GaAs branch, d_branch_, and the diameter of the corresponding trunk, d_trunk_, is shown in Figure 2. By precisely controlling the trunk diameter via lithographic techniques, nanometric precision (±1–2 nm) has been achieved for the branch diameters, extending into the subsequent Ge and SiGe segments. In fact, the diameter is related to the volume of the catalyst particle. Therefore, assuming that the contact angle between the Au particle and the NW remains consistent, thicker (thinner) trunks will result in thicker (thinner) branches.

**Figure 2:**
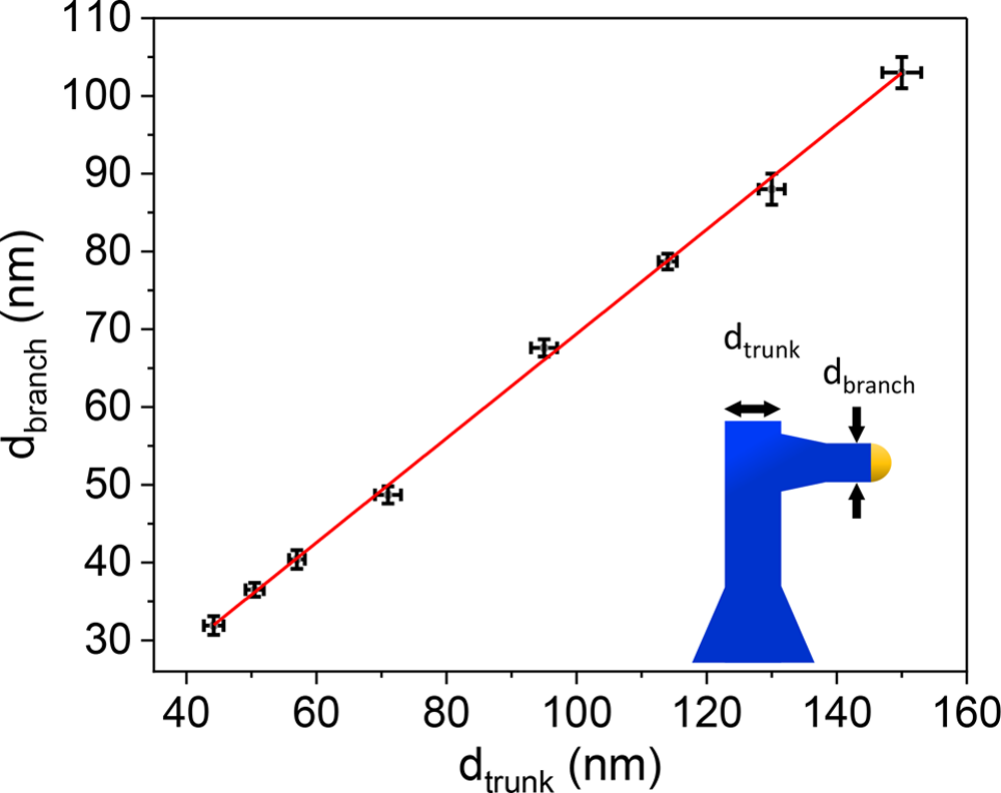
Linear relation between the diameter of the branch and the diameter of the respective trunk. Intercept = (2.3 ± 0.9) nm, slope = (0.671 ± 0.011).

The linear regression slope reveals that the diameter of the branches is approximately 67% of the trunk diameter, a trend consistent for all investigated diameters. Since no surface Au migration has been observed, the diameter reduction can be attributed to a combination of two factors: first, a decrease in the catalyst volume due to Ga depletion, and second, a change in the contact angle of the Au particle when transitioning from the {0001} to the  plane.

In fact, it is possible to prove that, for a constant catalyst volume, a decrease of 33% in the wire diameter would lead to an increase of 38% in the contact angle. While a contact angle of 90° for the trunk would result in a branch contact angle of 145°, ex-situ experimental measurements do not fully support such a large increase, giving contact angles between 105 and 135°(see Supporting Information). This suggests that also the reduction in catalyst volume due to Ga depletion likely plays a role in the observed diameter change. It is important to note that ex-situ measurements of the contact angle may differ from the actual contact angle present during growth. However, the arguments presented here rely on the comparison of contact angles measured under consistent conditions. Therefore, similar trends and results can be expected for both in situ and ex-situ measurements.

Precise control over the GaAs segment diameter also enables fine-tuning of the diameter of the subsequent portions of the branch, including the Ge and SiGe segments. In particular, a one-to-one relation has been found between the diameter of the GaAs and the one of the SiGe segments (additional data are provided in the SI).

After the growth of the GaAs branch, the system is cooled down before proceeding with the Ge growth. However, this transition poses challenges in producing straight Ge–GaAs heterostructured nanowires, caused by an effect not previously reported in earlier studies on III–V/IV heterostructures.^9−11^ In those works changes in growth direction at the interface have been attributed to the formation at the interface of a “compact nucleus” (or island), rather than to layer-by-layer growth, resulting in kinked segments. This difference in the growth dynamics is commonly attributed to the role of interface energies of the two materials. In our work, we identified an additional factor contributing to kinked wires, which resides in the morphology of the GaAs/catalyst interface.

Figures 3a and 3b show transmission electron microscopy (TEM) images of two interfaces between the GaAs segment and the catalyst particle, obtained by two different growth termination methods. In (a) both the flows of AsH_3_ and TMGa are interrupted, while in (b) only AsH_3_ is supplied for 4 min following the growth stop. Figure 3b displays a flat  facet, while Figure 3a shows an additional truncated  facet. It is found that straight end facets lead to straight nanowire growth, while truncated facets cause kinked growth. The presence of two distinct facets at the growth front, with different surface energies, may promote asymmetrical growth in the direction with the lowest energy of formation. It is thus important to understand and avoid the formation of the truncation.

**Figure 3:**
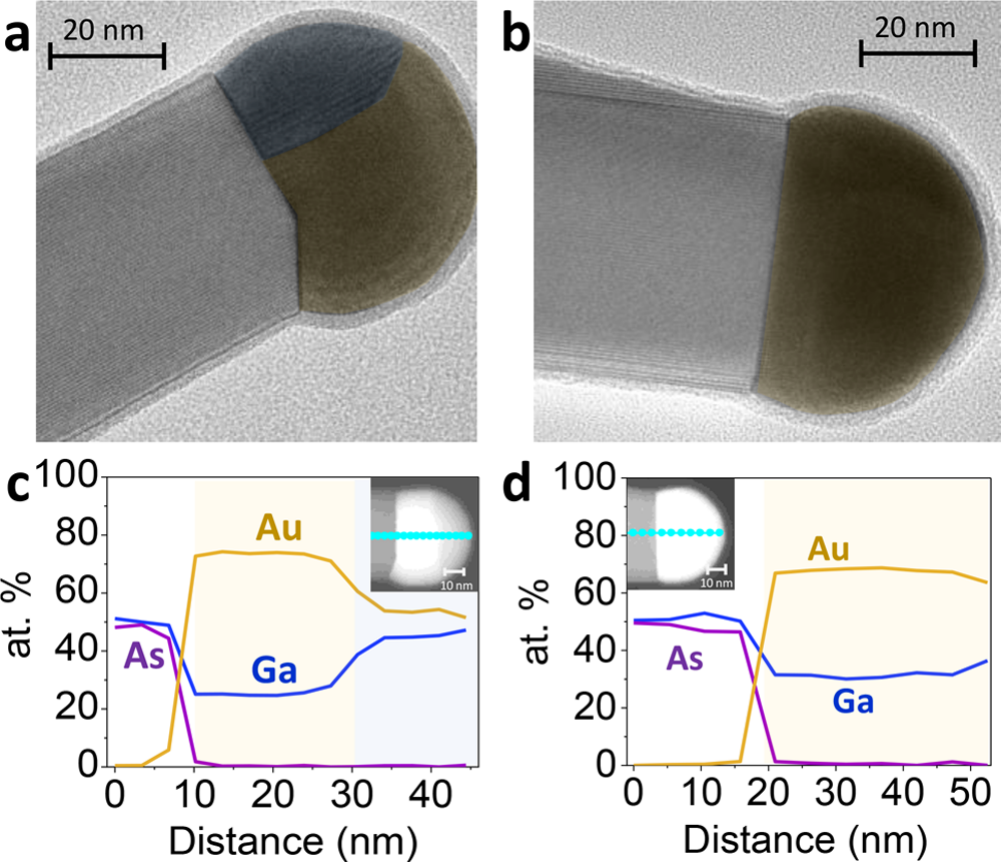
Different Au–Ga domains in the catalyst particle and their correlation with end facet truncation. a,b) False colored TEM micrographs of GaAs branches ending the growth without (a) and with (b) an additional AsH_3_ step. Two distinct Au–Ga phase domains are clearly visible in the first case. c,d) Compositional line profiles along the catalyst particle. When the AsH_3_ step is not applied, two domains, AuGa and Au_2_Ga, appear, correlating with facet truncation. A single Au_2_Ga domain is however observed when the extra AsH_3_ step is performed. Please note that Figures (a) and (c) are obtained from different wires.

Previous modeling of truncation formation as a function of contact angle^12,13^ demonstrates that for angles around 90° the end facet remains straight, whereas deviations from this range lead to truncation. Our ex-situ measurements of the contact angle for straight and truncated nanowires align well with these theoretical predictions, as detailed in the SI.

We speculate that the truncation might form during the cool-down process due to Ga dissolution from the wire and absorption into the catalyst particle. The As previously connected to Ga can now desorb in the chamber facilitated by the high growth temperature. This dissolution process leads to the truncated facet and an increase in the contact angle. It should be noted that the truncated facet is facing downward for all wires observed, implying that the polar nature of the  facet is relevant for the interface stability with the catalyst particle.

To avoid the truncation, we introduce the AsH_3_-only step for a few minutes at the end of the GaAs growth, in the absence of Ga precursor. In this scenario, AsH_3_ likely prevents this desorption process by saturating the environment and avoiding As desorption. This step reduces the Ga content within the catalyst particle, thereby lowering the contact angle.

The effectiveness of the AsH_3_ treatment at the end of the growth process is further confirmed by analyzing the composition of the Au droplet. Figures 3c and 3d show compositional profiles of the Ga content within the Au droplet after growth, acquired using energy-dispersive X-ray spectroscopy (EDX), comparing samples treated with and without the AsH_3_ step. Without AsH_3_, two Au–Ga eutectic domains are observed within the catalyst, also visible from the color contrast: one corresponds to the Au_2_Ga solid phase (with a percentage of 33% of Ga in the catalyst) and the other to the AuGa solid phase (with 50% of Ga in the catalyst). These phases align with the Au–Ga binary eutectic diagram.^14^ In contrast, after the AsH_3_ treatment, only a single Au_2_Ga phase is present, indicating a lower Ga content in the catalyst. This reduction in Ga justifies the smaller contact angles observed, resulting from the smaller particle volume.

The growth of Ge is then followed by a SiGe segment to complete the branch structure. Direct growth of SiGe on GaAs results in kinked SiGe nanowires, growing with a cubic crystal structure. This outcome is in agreement with previous observations on cubic III–V/III/V–SiGe heterostructures by Dick et al.^9^ To overcome this issue, a Ge buffer layer is introduced to modify the surface energies, enabling straight epitaxial growth.

TEM images of the full structure, as shown in Figures 4a–c, reveal an untapered branch growing straight along the  direction. A slight variation in diameter is noticeable between the different material segments, a phenomenon that has also been observed and described in Si/Ge heterostructures within cubic nanowires.^12,15,16^ The TEM images show an almost phase-pure wurtzite crystal structure, with few stacking faults propagating from the GaAs core up to the Au catalyst particle. No defects are created at the GaAs-Ge or Ge-SiGe interfaces, nor in the segments themselves. Additionally, there is almost no shell growth along the ⟨0001⟩ direction, which would favor cubic crystal growth (whereas growth in the  would result in a hexagonal structure), as observed in previous works,^5^ and would be easily detectable via TEM. This absence of shell material suggests that under the selected growth conditions the VLS mechanism dominates over VS growth, aided by the long diffusion length of Si and Ge adatoms on the nanowire surface.

**Figure 4:**
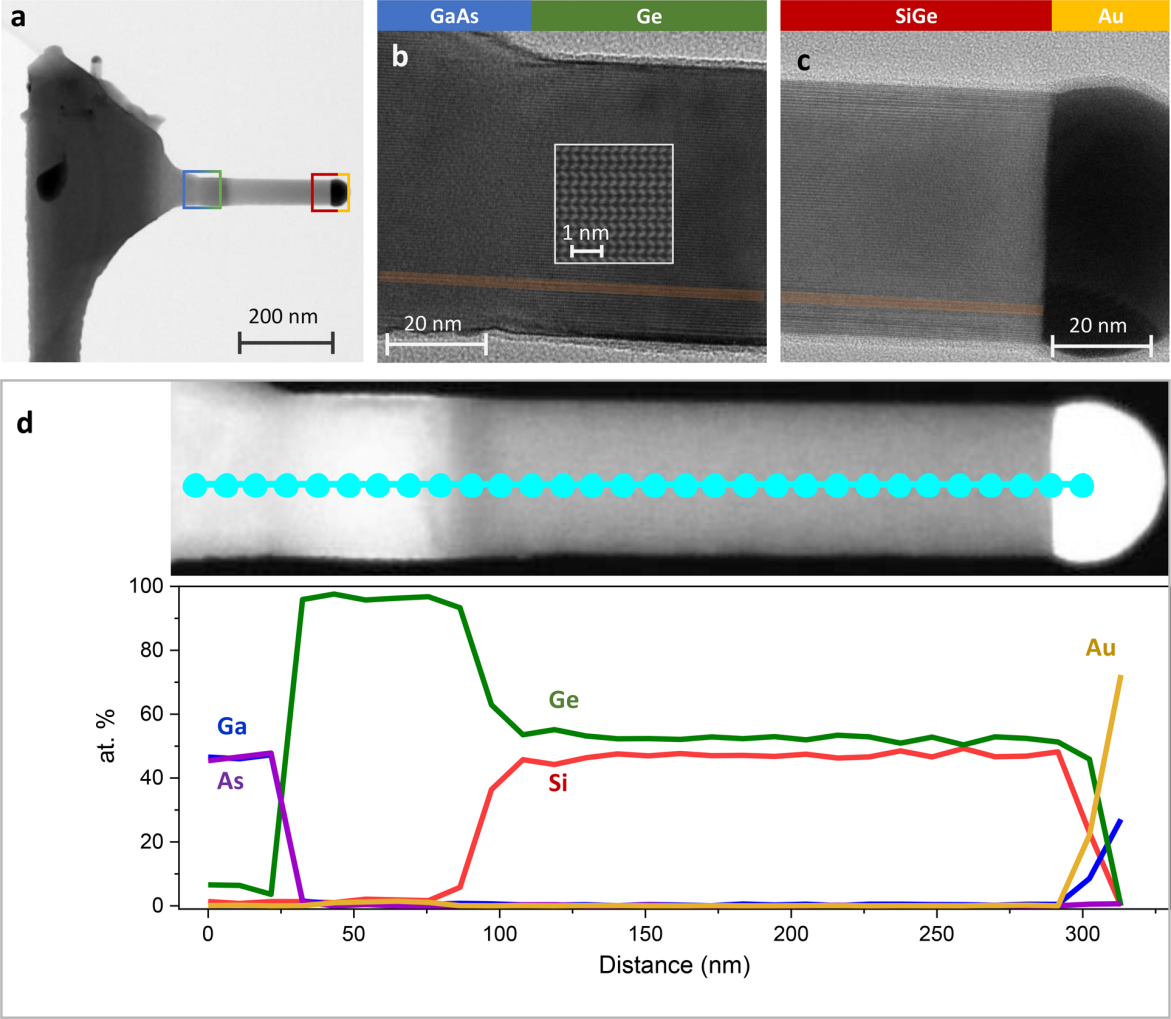
a) Bright-field STEM image of a SiGe branch performed along the  zone axis. b,c) More detailed bright field TEM images of the GaAs–Ge heterointerface and of the ending portion of the wire. A stacking fault is highlighted to appreciate the crystal structure homogeneity along the branch. d) Elemental composition profile of a full branch acquired using EDX.

A compositional profile of a branch is presented in Figure 4d. Although the primary focus of this work is not to characterize the interface, the profile provides an estimate of the abruptness of the heterointerfaces between GaAs and Ge, as well as between Ge and Si_0.5_Ge_0.5_. In both cases, the interface transition occurs over a distance of approximately 10–20 nm, which corresponds to 60–80% of the diameter of the analyzed branch. This demonstrates the relatively sharp heterointerfaces achieved in this structure.

Finally, to further investigate the growth dynamics, we analyze the growth velocity of the hexagonal Si_0.5_Ge_0.5_ branches across different diameters. Figure 5a shows the relation between the SiGe segment length and the growth duration for 6 different wire diameters. Growth times are varied between 30 min and 2 h. Within this range, for each diameter, a linear increase in NW length as a function of time is observed. Linear fits to the data for each diameter enables the calculation of growth rates and nucleation times. The latter represents the time required for the precursor gases to induce supersaturation in the Au droplet and initiate nanowire growth. Notably, no growth delay is observed upon transitioning from Ge to SiGe, as expected since the Ge and Si precursor flows have changed smoothly.

**Figure 5:**
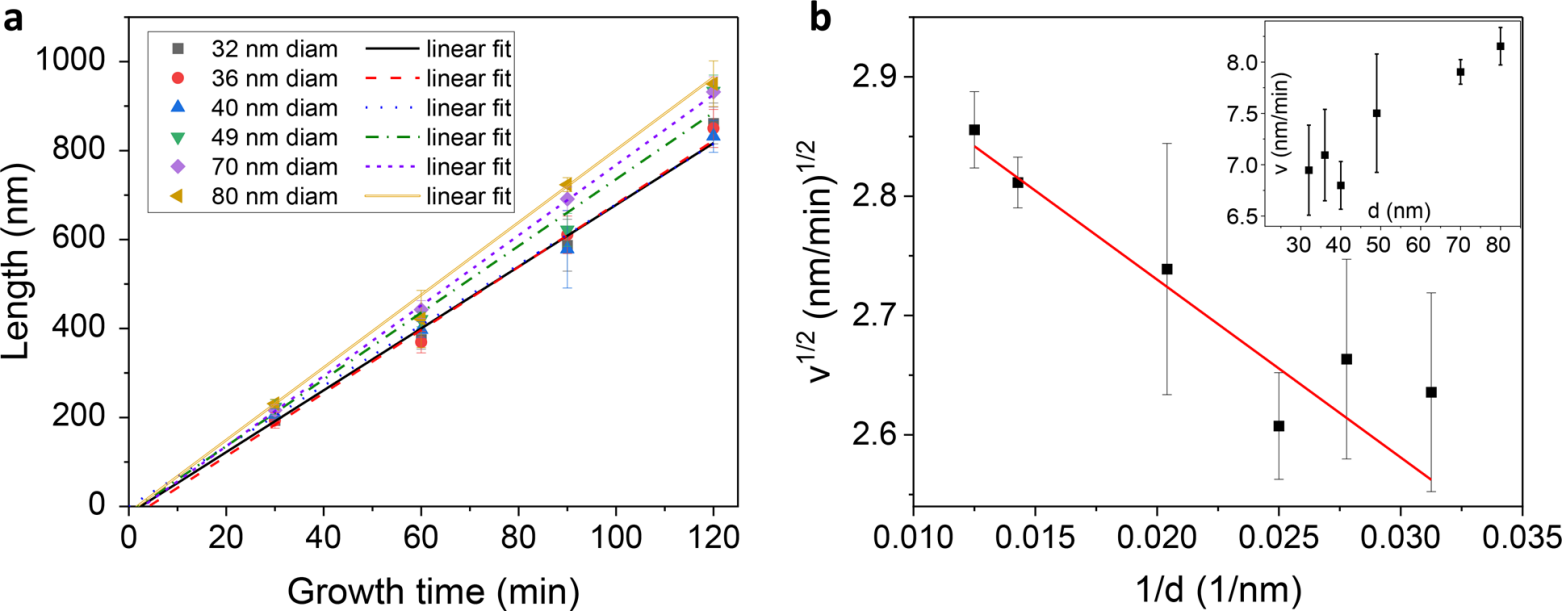
a) Plot and linear fit of the average length of the SiGe segments as a function of their growth time (fit details in the SI). The data are acquired for branches of different diameters. b) Plot and linear fit of the squared root of the growth rate as a function of the inverse of the branch diameter. The parameters extracted from the linear fit are intercept = (3.03 ± 0.04) , slope = (−15 ± 2) . The inset plot shows the growth rate as a function of the diameter.

The growth rates are plotted against the wire diameters, as depicted in the inset plot of Figure 5b. The observed increase in growth rate with increasing diameter suggests the influence of the Gibbs–Thomson effect. In fact, the high curvature of the particles may limit species absorption from the vapor phase, reducing the supersaturation within the liquid catalyst particle and reducing the growth rate.

Following the framework proposed by Dayeh et al.,^17^ based on the work of Givargizov,^18^ we can assume a quadratic dependency between the growth velocity and the supersaturation level, Δμ, which is defined as the chemical potential difference of Si/Ge atoms in the vapor and solid phase. In this context, it can be shown that the growth rate *v* can be described by the following expression:
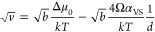
1Here μ_0_ is the supersaturation in the planar limit, *k* Boltzmann’s constant, *T* the temperature, Ω the atomic volume of the grown material, α_VS_ the average surface energy density of the NW facets,^19^ and *d* the wire diameter.

By plotting the squared root of the growth rate  as a function of the inverse of the diameter 1/*d*, we can fit the data linearly through eq 1. The result is shown in Figure 5b. Utilizing the atomic volume Ω = 2.13 × 10^–2^ nm^3^/atom for Si_0.5_Ge_0.5_, an average surface energy of α = 64 meV/Å^2^, and the growth temperature of 436 °C, we can derive key growth parameters of the system. Specifically, the system exhibits a cutoff diameter *d*_*c*_, where the supersaturation reaches zero and growth ceases, at *d*_*c*_ = (4.9 ± 0.7) nm. Additionally, the supersaturation value in the planar limit is Δμ_0_ = (110 ± 30) meV, and the kinetic coefficient is found to be *b*^1/2^α_VS_ = (7 ± 2)10^–9^ J/cm^3/2^/s^1/2^.

In conclusion, we have developed a novel growth method for hexagonal SiGe nanobranches, enabling precise control over both their diameter and length. We have revealed the conditions necessary for achieving straight epitaxial growth, as well as the relationship between the nanowire growth rate and diameter, which is explained by the Gibbs–Thomson effect. This new method introduces an unprecedented level of control over the morphology of hexagonal SiGe nanowires, fundamental for quantum applications and for the fabrication of light-emitting devices. In this regard, the presented Ge content of 50% can, in principle, be easily increased by adjusting the precursor ratio during growth.^1^ This could enable the formation of direct band gap SiGe, unlocking the optical properties of this material.

## Supporting Information Available

Detailed illustration of the growth process, alongside a comprehensive discussion on the role of nanowire base pyramids. This also includes measurements of the catalyst contact angle in the presence or absence of the AsH_3_ ending step, the linear relationship between the diameters of the SiGe and GaAs segments, fit parameters, and examples of common unsuccessful growth cases (PDF)
