# Supplementary material for: Dimension Control of Hexagonal SiGe Single Branched Nanowires
Source: Nano Lett. 2025 Mar 26;25(14):5741–6. doi: 10.1021/acs.nanolett.5c00267 (PMC11987063; doi:10.1021/acs.nanolett.5c00267)
Supplement: Supplementary file 1 — nl5c00267_si_001.pdf [file nl5c00267_si_001.pdf]

Supplementary Information: Dimension Control of Hexagonal SiGe Single Branched Nanowires

Denny Lamon,[†] Hidde A. J. van der Donk,[†] Marcel A. Verheijen,[†,‡] Marvin M. Jansen,[†] and Erik P.A.M. Bakkers[*,†]

†*Department of Applied Physics, Eindhoven University of Technology, 5600 MB Eindhoven, The Netherlands*

‡*Eurofins Materials Science Netherlands BV, 5656 AE Eindhoven, The Netherlands*

E-mail: e.p.a.m.bakkers@tue.nl

Methods

The nanowires are grown on GaAs $(1\,1\,1)_B$ substrate, in alow-pressure Aixtron Close Coupled Showerhead MOVPE reactor. Detailed wafer pre-processing steps prior to introduction into the reactor are described in previous work.[1] In summary, the GaAs(111) substrates are cleaned with diluted NH_4OH, spin-coated with PMMA-950K-A2, and patterned using electron beam lithography to create an array of square openings in the resist. Following development, a 6 nm layer of Au is deposited to form Au squares of the desired size after a lift-off process using PRS3000, acetone, and isopropanol. Finally, the sample undergoes a cleaning step with oxygen plasma and NH_4OH, making it ready for introduction into the reactor for growth.

Figure 1 illustrates the growth recipe for single-branched wires, presenting in detail the temperature profile and precursors flow variations. All temperature values refer to those measured by the thermocouple.

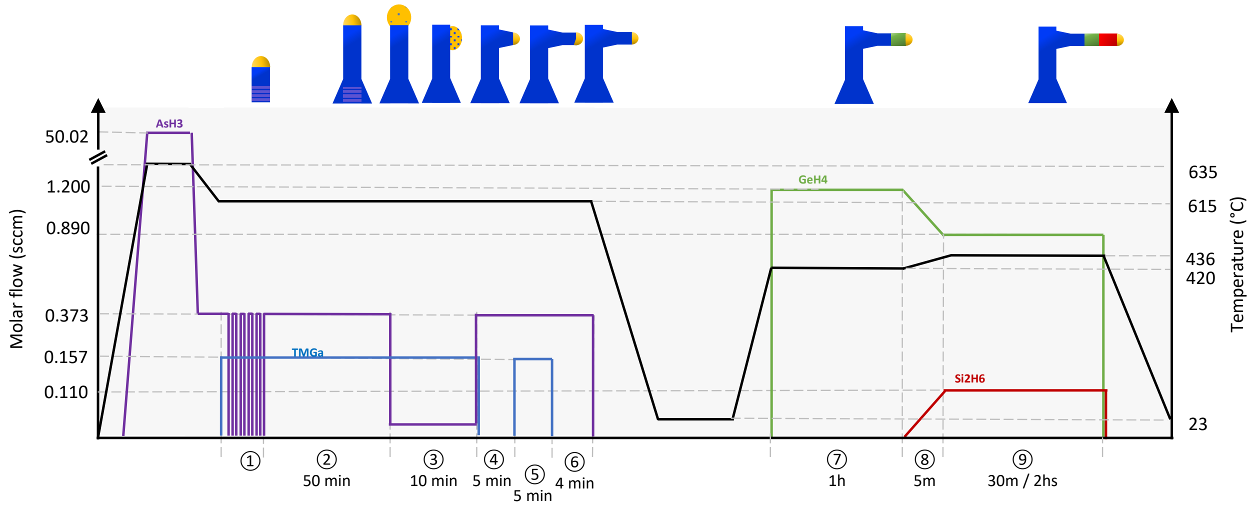

Figure 1: Precursors molar flow (AsH_3, TMGa, GeH_4, and Si_2H_6) and temperature profile over time during a typical SiGe nano-branch growth. Key growth steps are illustrated with a corresponding schematic above the plot.

Initially, the GaAs substrate undergoes a high-temperature treatment with an elevated AsH_3 flow to clean the GaAs surface. Growth conditions are then reached by reducing the temperature to 615°C and the molar flow of AsH_3 to 0.373 sccm. TMGa is then introduced with a molar flow of 0.157 sccm to promote nucleation of the initial GaAs NW layers. Following this, 30 Ga pulses (1) are performed by interrupting the flow of AsH_3 for 5 seconds every 10 seconds. The growth proceeds then under normal conditions for 50 minutes (2). A subsequent drastic reduction in the AsH_3 flow (3) for 10 minutes inflates the Au catalyst particles with Ga, leading to their destabilization. 5 minutes of AsH_3 only follow to deflate the catalyst droplet and form the GaAs flag (4). An additional 5 minutes of GaAs growth stabilizes the system, producing a small GaAs segment (5). TMGa flow is again interrupted for 4 minutes (6) to avoid the end facet truncation of the GaAs segment.

After this, the growth is stopped, cooling to room temperature and flushing the reactor.

For the subsequent Ge growth, a GeH_4 molar flow of 1.2 sccm is introduced after ramping the temperature to 420°C (7). The temperature is then further increased (8) up to 436°C, and the molar flows of GeH_4 and Si_2H_6 are changed respectively to 0.89 and 0.11 sccm, yielding a precursor ratio of $GeH_4/(GeH_4+Si_2H_6) = 80\%$. The growth is finally ended interrupting

the precursor flows and cooling the reactor under H_2.

Base pyramid generation

The formation of pyramids at the base of GaAs nanowires is still not completely understood and remains a stochastic phenomenon, showing unpredictable behavior that is difficult to control by simply adjusting the growth parameters. It is clear, however, that prolonged growth times lead to larger pyramids due to greater VS accumulation. Despite this, the precise onset of nucleation and the final size of these pyramids remain difficult to predict and manipulate.

We observed that pyramids at the base of the wires protect them, significantly improving the yield of standing wires during the inflation of the Au catalyst droplet. During this process only Ga precursor gas and hydrogen are introduced in the growth chamber, resulting in both the inflation of the Au droplets, but also in the formation of Ga droplets on the substrate. These particles can attach to the GaAs nanowire base, depleting As from the wire and consequently compromising their stability (figure 2d and 2e). This effect is highly mitigated when base pyramids are present, as they shield the wires from these harmful Ga droplets (figure 2c).

To induce pyramid formation also in the scenario of shorter growth duration, we leveraged a phenomenon observed by Peeters et al.,[2] wherein faster VS nucleation and growth occur near zincblende segments and stacking faults. It is well known how the crystallographic phase of the GaAs wires (weather wurtzite or zincblende) strongly depends on the contact angle of the catalyst particle with the NWs, which in turn depends on the particle's volume and the Ga content.[3,4] Based on this, we performed 30 Ga pulses interrupting the As precursor for 5 seconds and every 10 seconds (figure 2a). This resulted in the inflation of the catalyst, forming a zincblende segment of approximately 100 nm at the base, promoting the accumulation of material via VS growth. An example of a nanowire with an induced

pyramid is shown in Figure 2b.

This method granted highly controllable and reproducible pyramid formation, although the pyramids were sometimes smaller than those that formed stochastically during standard growth runs. Nonetheless, this process improved the overall homogeneity of the nanowire yield after Au destabilization, preventing large areas of the wafer from being devoid of nanowires.

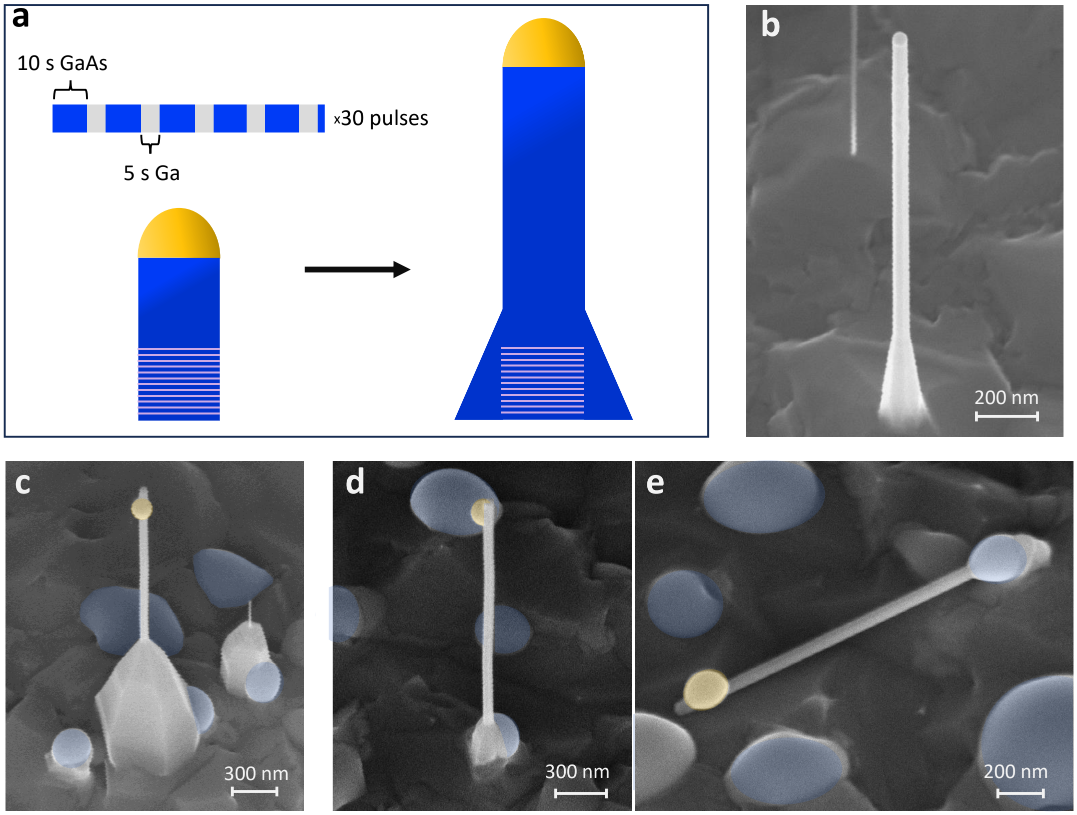

Figure 2: a) Schematic illustration of the pyramid formation process. During the growth of the NW, 30 pulses of Ga (each lasting 5 seconds, spaced 10 seconds apart) are introduced, leading to the formation of a zincblende segment at the base of the NW. This segment accumulates material via the vapor-solid mechanism. b) 30° tilt SEM micrograph of a GaAs nanowire with an induced pyramid. c) False-colored (Au in yellow, Ga in blue) SEM image of a wire with the base pyramid, highlighting the protective role of the latter in shielding the NW from Ga droplets formed on the sample surface. d-e) SEM micrographs of a nanowire without a pyramid in contact with a Ga droplet (d) and a fallen nanowire resulting from a Ga droplet interaction (e).

Larger views and yield considerations

Figure 3 presents large-area SEM images of nanowire fields after the main growth steps of the process. An estimate of the yield, defined as the ratio of successfully grown nanowires to the number of Au seed particles deposited on the substrate, is provided in the main text. It should be noted that this estimate may be influenced by sample-to-sample and intra-sample variations, as well as variations in the height of the GaAs trunks. However, such effects do not significantly affect the growth of the branches.

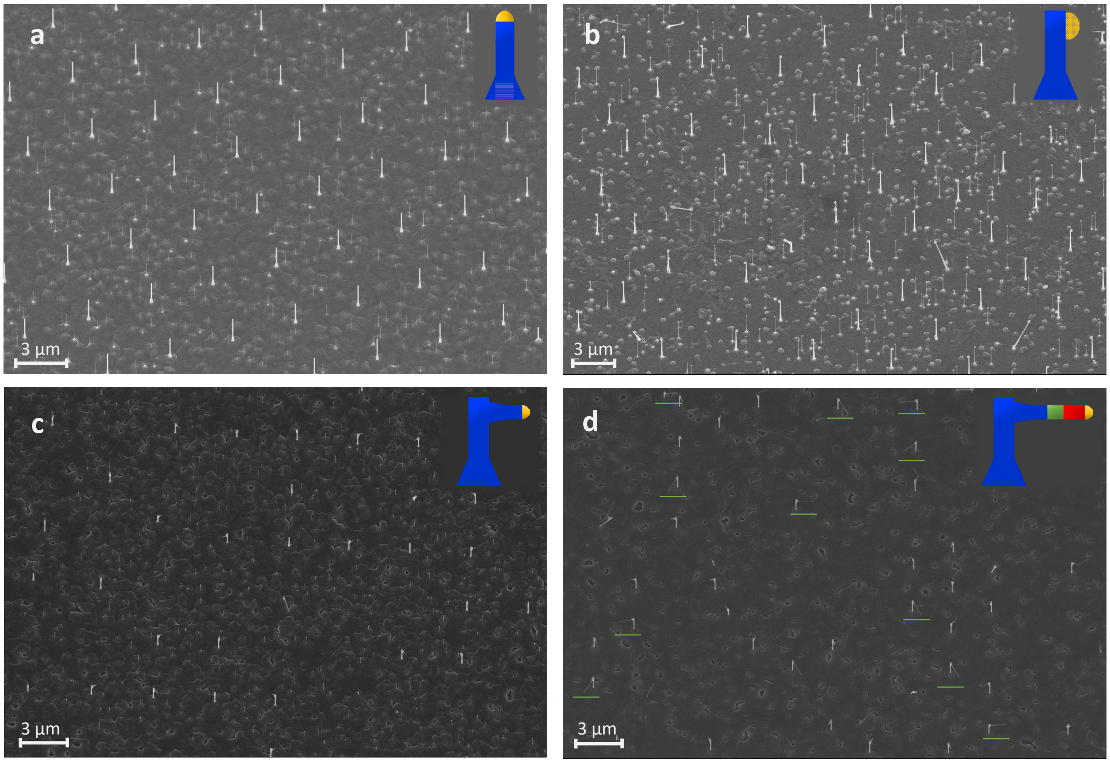

Figure 3: Large view SEM micrographs of the nanowire fields after the 4 main growth steps: a) growth of GaAs trunks, b) destabilization of Au particles, c) growth of GaAs segment, d) growth of full SiGe branch.

Catalyst contact angle and facet truncation

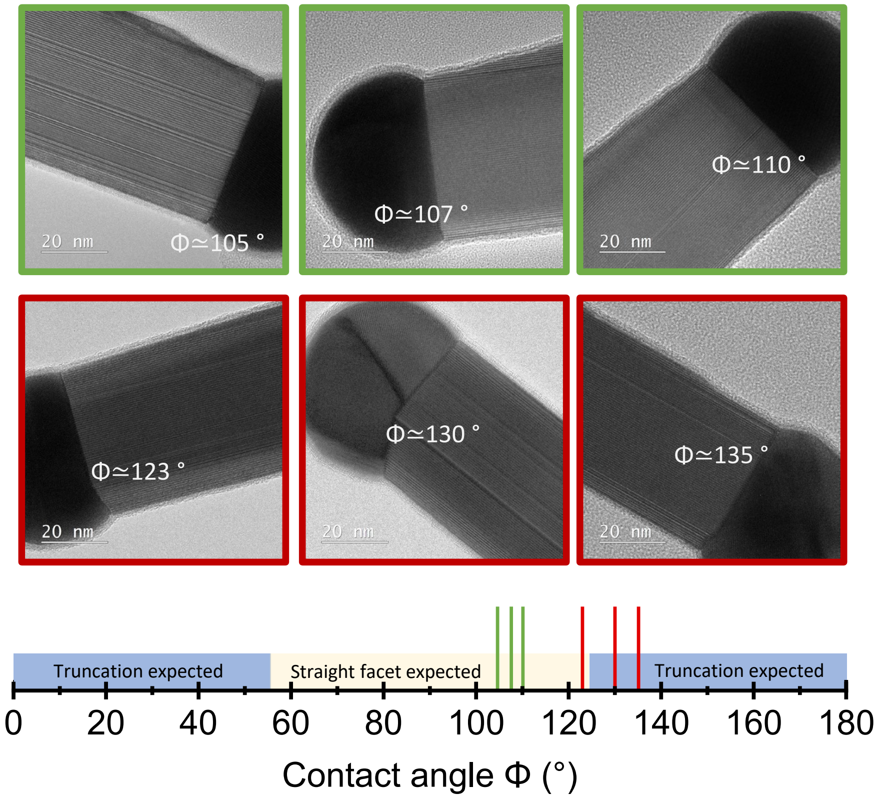

Figure 4: Ex-situ measured contact angles of the catalyst particles for both non-truncated end facets (first row, highlighted in green) and truncated end facets (second row, highlighted in red). The corresponding contact angles are reported as vertical lines in the bottom plot showing the ranges of expected truncated or straight end facets, as calculated by Jacobsson et al.[4]

Diameter of SiGe segment

In the main document, we presented the linear relation between the diameters of the GaAs segment and that of the trunk. Figure 5 demonstrates a similar linear relation between the diameter of the SiGe segment and the GaAs segment upon which it is grown. The slope, which is compatible with 1, and the intercept, near 0, confirm the precise control over the hexagonal SiGe segment, which can be achieved by controlling the diameter of the GaAs segment.

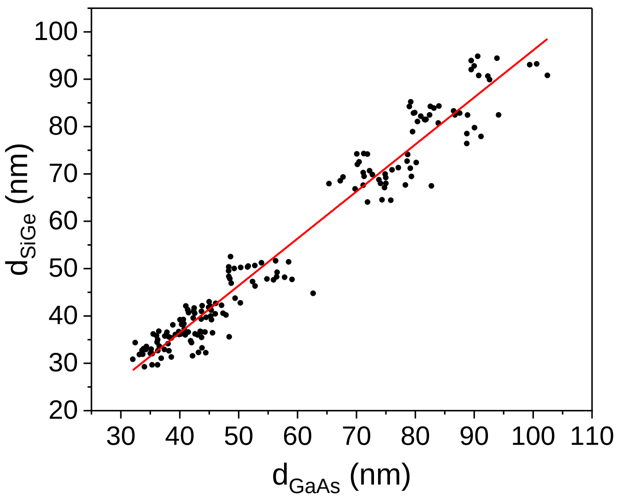

Figure 5: Linear regression of the diameter of the SiGe segment as a function of the one of the GaAs segment. Intercept = (-3.3 ± 1.0) nm, slope = (0.994 ± 0.017).

Linear fit details

Table 1: Fit parameters of the linear regressions presented in Figure 5a of the main document.

Diameter (nm)	Slope(Growth rate) (nm/min)	Intercept (nm)
32	6.9 ± 0.4	-17 ± 17
36	7.1 ± 0.4	-29 ± 27
40	6.8 ± 0.2	-1 ± 12
49	7.5 ± 0.6	-15 ± 36
70	7.9 ± 0.1	-23 ± 7
80	8.2 ± 0.2	-14 ± 10

Growth faults

The growth of single SiGe branched nanowire occasionally deviates from the desired straight hexagonal configuration, resulting in defective growth modes. In Figure 5, we illustrate the two main growth mechanisms that deviate from the intended growth regime.

One such mechanism involves backward growth at the GaAs-Ge interface, where instead of growing axially, the branch extends along the surface of the GaAs flag. This mechanism could be caused by not straight GaAs end facets after the extra AsH_3 step, or by defects

that lead to the wetting of a side facet by the Au particle. In these cases, growth follows a cubic-backward orientation rather than the intended hexagonal-outward one.

Another observed growth anomaly, shown in Figures 5c and d, is a switch in the crystallographic growth direction from $\langle 1\,0\,\bar{1}\,0 \rangle$ to $\langle 1\,1\,1 \rangle$ during the GaAs branch development. When this occurs, the growth no longer copies the crystal structure and instead enters a mixed-phase regime for GaAs, resulting then in cubic Ge and SiGe branches that grow along the least energetic direction. This shift in crystallographic orientation is easily identifiable by the angle formed between the branch and the trunk, which is approximately 70°, in contrast to the expected 90°angle characteristic of hexagonal epitaxial growth.

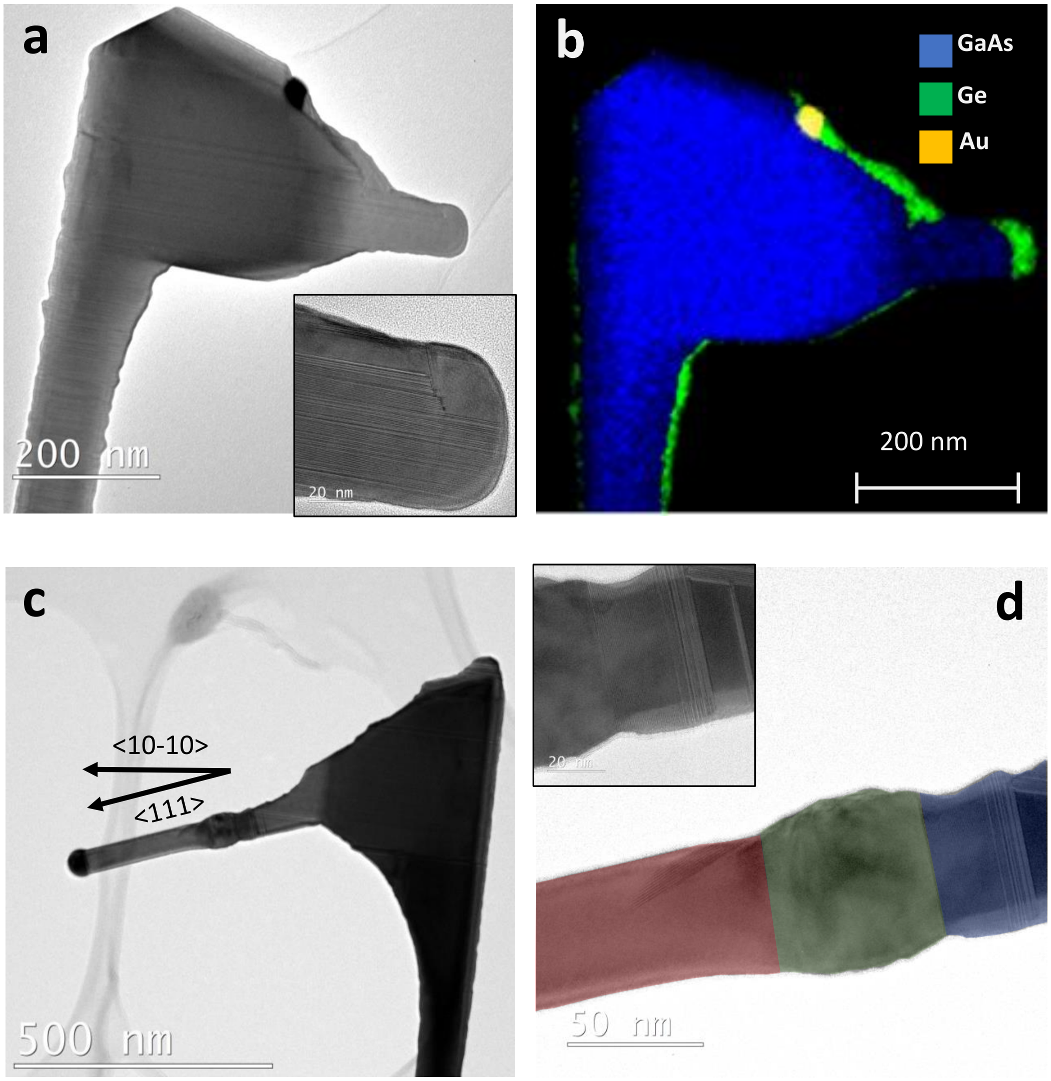

Figure 6: a) BF-STEM image and detail of the heterostructure and (b) EDX scanning map of a backward-grown Ge branch. The growth initiates along the outward direction and then proceeds backward over the surface of the GaAs flag. c) BF-STEM image and detail of the heterostructures (d) of a GaAs branch changing the crystallographic growth direction from hexagonal $\langle 1 0 \bar{1} 0 \rangle$ to cubic $\langle 1 1 1 \rangle_{\mathrm{B}}$. The subsequent Ge and SiGe segments are copying the cubic crystal stacking.

References

(1) Peeters, W. H. J.; Vettori, M.; Fadaly, E.; Danescu, A.; Mao, C.; Verheijen, M. A.; Bakkers, E. P. A. M. Understanding the Onset of Uncontrolled Polytypism During the Au-Catalyzed Growth of Wurtzite GaAs Nanowires. *Physical Review Materials* **2024**,

8.

(2) Peeters, W. H. J.; van Lange, V. T.; Belabbes, A.; van Hemert, M. C.; Jansen, M. J. J.; Farina, R.; van Tilburg, M. A. J.; Verheijen, M. A.; Botti, S.; Bechstedt, F.; Haverkort, J. E. M.; Bakkers, E. P. A. M. Direct Bandgap Quantum Wells in Hexagonal Silicon Germanium. *Nature Communications* **2024**, *15*.

(3) Glas, F.; Harmand, J.-C.; Patriarche, G. Why Does Wurtzite Form in Nanowires of III-V Zinc Blende Semiconductors? *Physical Review Letters* **2007**, *99*, 146101.

(4) Jacobsson, D.; Panciera, F.; Tersoff, J.; Reuter, M. C.; Lehmann, S.; Hofmann, S.; Dick, K. A.; Ross, F. M. Interface Dynamics and Crystal Phase Switching in GaAs Nanowires. *Nature* **2016**, *531*, 317–322.
